# Profiling the Spectrum of Headache Disorders on 440 Breast Cancer Patients: Highlights on Clinical and Pathological Mechanisms

**DOI:** 10.3390/biomedicines11041059

**Published:** 2023-03-30

**Authors:** Mariya Boyanova Ilieva, Paola Tiberio, Rosalba Torrisi, Jacopo Lanzone, Vittorio Di Piero, Armando Santoro, Alessandro Viganò, Rita De Sanctis

**Affiliations:** 1 Department of Biomedical Sciences, Humanitas University, 20072 Pieve Emanuele, Italy; mariya.ilieva@humanitas.it (M.B.I.); armando.santoro@cancercenter.humanitas.it (A.S.); rita.de_sanctis@hunimed.eu (R.D.S.); 2 Medical Oncology and Hematology Unit, IRCCS Humanitas Research Hospital, 20089 Rozzano, Italy; paola.tiberio@cancercenter.humanitas.it (P.T.); rosalba.torrisi@cancercenter.humanitas.it (R.T.); 3 Neurorehabilitation Department, IRCCS Salvatore Maugeri Foundation, Institute of Milan, 20138 Milan, Italy; jacopo.lanzone@icsmaugeri.it; 4 Department of Human Neurosciences, Sapienza University of Rome, 00185 Rome, Italy; vittorio.dipiero@uniroma1.it; 5 IRCCS Fondazione Don Carlo Gnocchi, 20148 Milan, Italy

**Keywords:** neoplasm, headache, tension-type headache, disability, breast cancer, migraine, hormone receptor expression, HER2, menopause, peripheral neuropathy

## Abstract

Although widely studied, the association between migraines (M) and breast cancer (BC) risk remains evasive. In this prospective single-center study, 440 early or locally advanced BC patients were enrolled at IRCCS Humanitas Research Hospital. Clinical and demographical data were collected. Those who suffered from headaches were evaluated with the International Classification of Headache Disorders. M was found to be significantly more prevalent in BC patients: 56.1% versus an expected prevalence of 17% in the global population. M patients showed a higher risk of having stage II or III BC than stage I, which was more frequently found in the non-headache population. Interestingly, the frequency of headache attacks was positively correlated with estrogen (*r* = 0.11, *p* = 0.05) and progesterone (*r* = 0.15, *p* = 0.007) expression, especially in patients with migraine without aura. The higher the expression of hormone receptors in BC, the higher the headache frequency. Moreover, patients suffering from headaches showed an overall earlier onset of BC. Our findings challenge the idea of a net preventive role of M on BC, suggesting a rather complex interaction in which M mostly influences some BC subtypes and vice versa. Further multi-center studies with extended follow-up are needed.

## 1. Introduction

Breast cancer (BC) is a very heterogeneous disease with a multifactorial etiopathogenesis characterized by a strong hormonal influence [1]. This is easy to figure out since the breast is a gland indirectly controlled by the hypothalamic–pituitary–ovary axis. Nowadays, it is estimated that about one in eight women will receive a BC diagnosis in their lifetime [2]. On the other hand, a deeper understanding of the molecular mechanisms underlying BC development and progression has led to the development of increasingly accurate prognostic tests and new effective drugs, thus leading to an increase in the overall survival of BC patients [2,3,4]. As a result, the number of BC survivors is continuously increasing, and knowing the complexity of BC survivorship is essential for adequate patient management.

Recently, a scientific interest covering possible interactions between migraine (M) and BC has been developed. Although the concordance among studies is incomplete, in some large epidemiological investigations, M seems to be associated with a lower overall risk [5,6,7,8,9,10,11] and, in some cases, a less aggressive histopathological BC phenotype with a consequent better prognosis [11]. However, this relationship has been found only in case–control studies and not in cohort ones [10,12,13,14], suggesting that it could be either a causal or a spurious association due to the high prevalence of these two diseases in the global population. Indeed, tension-type headaches (TTH) and M are the second and third most common disorders worldwide [15,16], respectively, and BC is the most common neoplasm in women [3,17]. Therefore, these conditions could often overlap among them by simple chance. This is even more probable due to the long course of headache diseases: M generally starts in infancy/adolescence, and its prevalence rises through the decades, at least up to the age of 65 years, while TTH generally has a later onset but it lasts until an older age [18,19,20,21].

Another possible confounding factor is that the majority of the published studies were made on large epidemiological multicenter registries with virtually no control over the criteria used for M diagnosis and often lacking information on BC molecular subtypes and management.

To clarify this issue, we performed a prospective study by applying the third version of the International Classification of Headache Disorders (ICHD-3) [22] to obtain a specific headache diagnosis, together with clinical and pathological data of BC and specific clinical features of headache.

## 2. Materials and Methods

### 2.1. Participants

We prospectively recruited patients with BC diagnosis attending the Breast Unit of IRCCS Humanitas Research Hospital from May 2021 to June 2022. Inclusion criteria were age > 18 years, histologically confirmed diagnosis of invasive BC, stage I to III BC, current follow-up at the specialized BC outpatient clinics, and acceptance to participate in the study. We excluded patients with incomplete data on either BC or headaches, patients with advanced disease (i.e., BC distant metastases or synchronous metastatic second primary tumor), or other concomitant invasive malignancies, as well as all patients diagnosed with a secondary form of headache. The research protocol was approved by the local Ethical Committee (Independent Ethical Committee IRCCS Humanitas Research Hospital, protocol number ONC/OSS-14/2021, date of approval: 18 May 2021). Written informed consent to the use of clinical data for scientific purposes was provided by all patients at the time of the first visit to IRCCS Humanitas Research Hospital. All procedures were conducted according to the principles of the Helsinki Declaration.

### 2.2. Data Collection

Demographic data, comorbidities, and BC risk factors (e.g., body mass index (BMI), smoking) were collected together with menopausal status at BC diagnosis and study enrollment, including date of last menstruation and hormone replacement therapy use. We also recorded the histopathological diagnosis for each recruited patient, together with therapeutic data (surgery, chemotherapy regimens, radiotherapy, and endocrine therapies each patient underwent), clinical response achieved, relapse date (if any), and any treatment-induced adverse events (e.g., taxane-induced neuropathy). Furthermore, data on main comorbidities were collected according to 6 major categories (i.e., metabolic, cardiovascular, psychiatric, gastrointestinal, ophthalmological, and neurological disorders), which could play a role in the development of headaches.

A headache diagnosis was obtained by a custom-made questionnaire based on ICHD-3 criteria for the most common forms of headache. In particular, the survey included criteria aimed at reaching out to the second level of a headache diagnosis, limited to M without aura (MWOA), M with aura (MWA), chronic M, as well as infrequent, frequent, and chronic TTH. Due to the hormonal link connecting BC and M, criteria per pure- and menstrually related M, with and without aura, were included.

The results of the questionnaires were checked by a neurologist trained in headaches to assess the reliability of responses and formulate a diagnosis. In the case of uncertainty between probable M and TTH, the latter was preferred, as recommended by ICHD-3. Questionnaires in which a diagnosis was not possible were discarded.

As covariates of interest, age of headache onset, number of monthly headache days, use of analgesics/triptans, use of preventive treatments for headache, as well as the status of activity of the headache (headache was considered active if the patient had any attack within one year prior to the recruitment) were recorded [23].

### 2.3. Statistical Analysis

Data distribution was tested by the Shapiro–Wilk’s test. Parametric or nonparametric tests were used accordingly. Data were presented as mean and standard deviation (SD), or percentage or median and range when needed. Due to the normal distribution, one-way ANOVA was used to test continuous variables among multiple groups, with Bonferroni post hoc correction for multiple comparisons. Chi-square or Fisher’s exact test (including the Fisher–Freeman–Halton’s extension) were used for categorical variables according to the number of subjects per cell. The significance level was set at *p* < 0.05 after proper correction. For the correlation analysis, Pearson correlation or Spearman’s rank correlation were used according to data distribution. Discriminant function analysis (DFA) was used as a multivariable analysis method to investigate whether systemic BC treatments could have affected the prevalence of headaches in the sample. In brief, DFA is a statistical tool used to group cases based on selected covariates related to the outcome of interest. It provides a model listing the variables associated with the outcome in descending order of their impact. DFA has been widely described elsewhere [24,25,26,27,28]. Statistical analyses were performed with STATISTICA, v.7 (StatSoft Inc., Tulsa, OK, USA).

## 3. Results

A total of 440 consecutive BC patients were enrolled in the study during outpatient visits, from which 324 complained of suffering or having suffered from headaches in the past and were, therefore, interviewed via questionnaires to obtain information on the characteristics of their headache after the discard of 11 questionnaires because of the inability to reach a headache diagnosis.

### 3.1. BC Patients’ Characteristics

Demographic, clinical, and pathological characteristics of BC patients are represented in Table 1. Specifically, all recruited patients were female, with a mean age at BC diagnosis of 53.8 years (SD ± 12.1). The median ECOG PS (Eastern Cooperative Oncology Group Performance Score) was 0, ranging from 0 to 3. The average BMI was 24.7 kg/m^2^ (SD ± 4.9). The menopausal status of the patients was recorded both at the time of BC diagnosis as well as at their last outpatient visit (thus considering the potentially induced menopausal status, either for therapeutic purposes through endocrine therapy or as chemotherapy-induced amenorrhea). The majority of women were already in menopause at the time of BC diagnosis and in the span from the diagnosis to the last visit, the percentage of women in menopause further increased. Regarding comorbidities, cardiovascular and metabolic (including dyslipidemia, diabetes, and hyper/hypothyroidism) diseases were the most frequent (either alone or in addition to other comorbidities), accounting for 44.3% (195/440) of the patients. Other comorbidities (84/440, 19.1%) included psychiatric, gastrointestinal, ophthalmological, and neurological disorders.

Regarding BC characteristics (reported in Table 1), the most common histologic subtype was NST (no special type) with a total of 347/440 patients (78.9%), followed by lobular BC with 11.8% (52/440), mixed histology (e.g., ductal and lobular) with 4.5% (20/440), and other less common histotypes (e.g., mucinous, micropapillary, cribriform, tubular, medullary, and papillary) with 3.9% (17/440). The majority of patients presented a moderately or a poorly differentiated BC with a Nottingham score of G2 (271/440, 61.6%) or G3 (132/440, 30%), respectively. Most of the patients had an early stage diagnosis, with 46.4% (204/440) at stage I, and 43.4% (191/440) at stage II, while stage III was only diagnosed in 10.2% (45/440) of BC patients. Considering the molecular subtype, according to the literature, the Luminal ones were the most frequent. Specifically, 42.5% of BC patients (187/440) presented Luminal A cancers, 28.9% (127/440) Luminal B Human Epidermal Growth Factor Receptor 2 (HER2) negative ones, 13.6% (60/440) Luminal B HER2 positive neoplasms, 6.6% (29/440) the hormone receptor (HR) negative HER2 positive subtype, and 8.2% (36/440) were diagnosed with triple-negative BC (TNBC). For one woman, BC was first diagnosed in 1997; thus, we did not have HER2 immunohistochemistry staining intensity but only HR ones. Of our entire population, only 52 patients underwent genetic counseling and testing for BRCA1 (BReast CAncer gene 1) and BRCA2 mutations (BReast CAncer gene 2). Of these, eight patients carried BRCA1 mutation, 4 BRCA2, while the remaining 40 were BRCA wild-type.

In relation to therapy, most patients received adjuvant therapy, 84.3% (371/440), while 14.1% (62/440) were also treated with neoadjuvant therapy. According to biological subtype and risk factors, 207/440 (47.0%) patients received chemotherapy treatments (Table 2), with the majority of them receiving anthracyclines (typically, anthracyclines plus cyclophosphamide, AC) followed by taxanes (69.1%, 143/207). Anti-HER2 agents (i.e., Trastuzumab, Pertuzumab, and TDM-1 (trastuzumab emtansine)) were given to 91/440 patients (20.7%) with HER2-positive BC. In total, 365/440 HR-positive BC patients were treated with hormonal therapy, and specifically, 131/365 (35.9%) were treated with tamoxifen, 173/365 (47.4%) received aromatase inhibitors (AI; Anastrozole, Letrozole, or Exemestane), 57/365 (15.6%) were under the regimen of two years of adjuvant therapy with tamoxifen which was then switched to AI for the next three years until the completion of a total of five years of hormonal therapy, and 4/365 (1.1%) received other hormonal therapies. Moreover, 122/440 (27.7%) patients were treated with luteinizing hormone-releasing hormone (LH-RH) analogs to pharmacologically induce menopause, including five TNBC patients for gonadal protection. Finally, 324/440 patients (73.6%) received postoperative radiotherapy. Of note, 51/157 (32.5%) women treated with taxanes developed peripheral neuropathy, whereas of the 432 BC patients treated with chemotherapy and/or hormonal therapy, 28.9% (125/432) and 1.4% (6/432) reported arthralgia and allodynia, respectively, regardless of therapy type.

### 3.2. Headache Profiles

Out of 440 patients, 319 reported suffering or having suffered from headaches, while 121 reported less than five episodes of headaches in their life of any kind and were therefore considered as patients without headaches (NONH). Out of 319 patients, the majority of patients were diagnosed with MWOA, while MWA was found in about 18.5% of cases, and about one patient out of five (22.6%) had TTH (Table 3). Of note, 49.1% of MWA, 54.3% of MWOA, and 47.2% of TTH reported headache onset beyond the second decade of life. In a significant fashion, more than half of the migraineurs reported their headache to be menstrually related, with 50.8% of MWA and 59.6% of MWOA, while, on the other hand, only 25% of the patients with TTH reported it to be somehow menstrually related (*p* = 0.001) (Table 3).

Overall, menopause influenced the headache pattern in different groups of patients (*p* = 0.008): 43.9% of patients with MWA reported a reduction in headache frequency, while in 17.5%, the frequency increased, and in 33.3%, the frequency did not change. In MWOA patients, the percentage of improvement with menopause was similar (41.1%), while less than half of patients with MWA (8.1% vs. 17.5%) had a worsening of headaches; in 39.5%, frequency did not change. Menopausal status showed less influence for TTH as 66.7% of the patients reported no alteration, 26.1% had an improvement, and 5.8% worsened (Table 4).

### 3.3. Effects of BC Therapies on Headache

BC diagnosis and treatment generally did not affect headache burden. In fact, the majority of patients reported that the number of headache days did not vary significantly at the moment of the BC diagnosis (*p* = 0.17), especially when we considered headache subgroups since headaches remained stable in 80.7% of MWA, 90.4% of MWOA, and 83.1% of TTH (Table 4). Neither systemic therapies for BC, such as chemotherapy, affected headaches (*p* = 0.37) for all three headache types (Table 4). Interestingly, hormonal therapy also had no relevant impact on headache frequency, contrary to common expectations (*p* = 0.24) (Table 4). Of note, the only BC therapy that affected the headache burden was locoregional radiotherapy (*p* = 0.02). Despite the small number of patients who changed their headache pattern during radiotherapy, a significantly higher portion of patients with MWA reported to have changed their headache status (either as improvement or worsening of headache frequency) at the moment of radiotherapy, compared with MWOA and TTH. In fact, about 7.0% of MWA patients experienced headache worsening vs. 2.2% of MWOA patients, while 3.5% of patients improved in MWA vs. none of the other two groups (Table 4). Moreover, to strengthen our finding, we conducted a DFA to unveil a possible association among specific headache types and the allocation to a particular BC treatment. However, the DFA model was not significant (F (3.43) = 1.23; *p* = 0.29), thus discarding the chance that BC therapies could have influenced our results.

### 3.4. Comorbidities Interplay and Adverse Event Rate

Overall, patients with headaches did not differ from NONH in terms of comorbidities: neurological (*p* = 0.67), gastrointestinal (*p* = 0.54), psychiatric (*p* = 0.20), ophthalmologic (*p* = 0.95), and metabolic (*p* = 0.31) fields were not significantly different among the two groups, while only a slight trend was found for cardiovascular comorbidity (*p* = 0.07), which was more represented in patients with headaches.

Among adverse events, we only found a higher rate of peripheral neuropathy due to chemotherapy with taxanes in patients suffering from MWOA and TTH compared to NONH patients (*p* = 0.048), while this was not true for MWA patients.

### 3.5. Relationship between BC and M

We did not find a specific association between headache diagnosis and BC subtypes, being the percentage of HR-positive (either Luminal A and Luminal B), HER2 positive, and TNBC resembling the proportion of BC patients without headaches similar to those with MWA, MWOA, and TTH (*p* = 0.64). By contrast, M patients showed a higher risk of having a diagnosis of stage II or III BC rather than stage I, which was more frequently found in NONH patients. Interestingly, MWA patients were more frequently diagnosed at stage II, while the risk of being diagnosed at stage III was equally higher in MWA and MWOA (Table 5).

**Figure 1:**
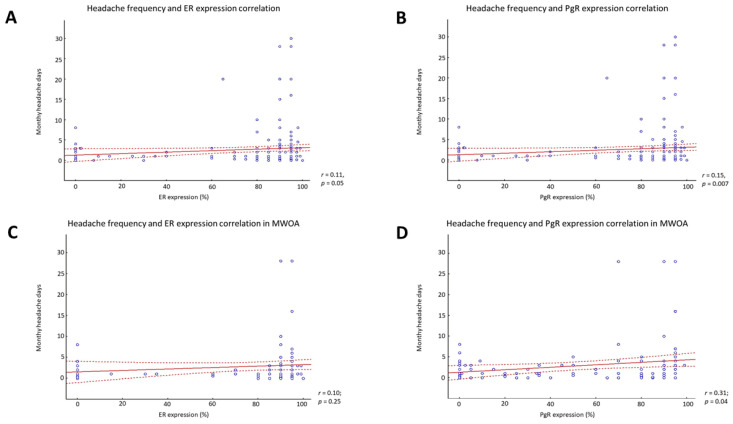
Headache frequency in relation to ER and PgR expression in all headache subgroups (**A**,**B**), respectively and in MWOA (**C**,**D**), respectively. Blue circles represent individuals (subjects may be overimposed). The continuous red line represents the correlation line and the dotted red lines the ± 95% CI of the correlation value.

When considering the burden of headaches in both M and TTH, we found that the frequency of headache attacks was positively correlated with female HR expression in BC malignancies, although less for estrogen (ER) (*r* = 0.11, *p* = 0.05) than progesterone (PgR) (*r* = 0.15, *p* = 0.007) (Figure 1). When analyzing different headache types separately, we found that this correlation was maintained only in MWOA between monthly headache days and PgR receptors (*r* = 0.31; *p* = 0.04). MWOA, MWA, and TTH patients did not differ in the expression of either HR overall or ER (F(6, 846) = 0.26018, *p* = 0.95).

**Figure 2:**
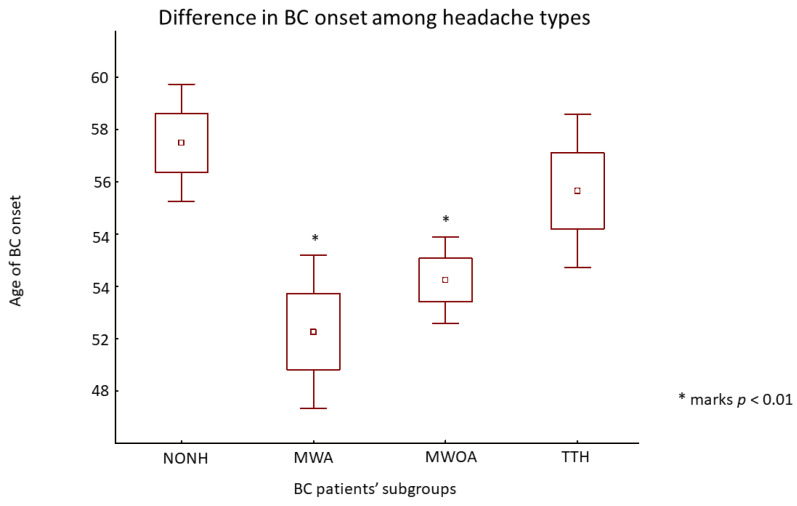
Age of BC development in relation to headache diagnosis.

Patients suffering from headaches showed an overall earlier onset of BC diagnosis compared to NONH, who had a mean age at BC diagnosis of 57.5 years ± 12.2 (F(3, 431) = 7.22; *p* = 0.0001) (Figure 2). In detail, MWA showed the earliest onset of BC (50.3 years ± 11.2) (*p* = 0.0008, Bonferroni corrected), followed by MWOA (52.2 years ± 11.44) (*p* = 0.001, Bonferroni corrected), and then TTH (55.6 years ± 12.5, *p* = 1.00 Bonferroni corrected) being not significantly different from NONH.

To date, 40 out of 440 patients have experienced a BC recurrence, with a relapse rate of 10% with no significant differences among each type of headache (*p* = 0.37): in detail, of the relapsed patients, 3 were MWA, 20 MWOA, 4 TTH, and 13 NONH.

## 4. Discussion

The most striking result was that the prevalence of MWA and MWOA in our sample was much higher than expected from the general population concerning female prevalence. We found that 56.1% of our 440 BC patients were diagnosed with M (59 MWA + 188 MWOA). This prevalence is higher than the expected M prevalence of 17% in the general population and 18.6% for women aged 20–64 years old [29]. Similarly, the prevalence of MWA was 13.4% compared to about 5.3% in women in the global population [30]. On the other hand, the proportion of TTH seems lower than expected, about 17% versus an expected prevalence of 26% in updated reports [29]. This result seems to be consistent with our previous pilot study [31]. However, other independent replicative studies are required to confirm these findings. Finally, the proportion between MWA and MWOA in our sample (MWA:MWOA = 1:4) appears to reflect those reported in M studies [30], suggesting an actual high prevalence of M in this population. This datum seems to be stable from our previous study on this subject, i.e., 13.4% in the present study versus 14.0% in the previous pilot study [31].

During recent decades, there have been many studies regarding the possible correlation between BC and headache disorders, specifically M [5,6,7,8,9,10,11,12,13,14,23,32]. BC is known to be the most frequent malignancy among women worldwide [3,17]. On the other hand, M and TTH rank as the third and second most common neurological disorders, respectively [15,16]. Therefore, we could expect a significant overlap just by chance. However, several publications have shown a possible beneficial effect of M on BC, suggesting a lower risk or a less aggressive BC profile, although some methodological bias could have influenced the outcome. Indeed, the protective effect of M on BC was highlighted only in case-control studies and not in cohort ones [10,12,13,14,23,32] and, to date, it is still an open issue. Plus, there is no clear explanation of the underlying mechanisms of how M could act as a BC protective factor.

In the present study, we aimed to expand the design of a prior pilot study we conducted a few years ago [31] by studying a sample size large enough to be informative. Furthermore, since the enrollment of both localized and advanced BC patients in our pilot study was a critical point for the result interpretation, we decided to exclude advanced BC disease from this project. We recruited a total of 440 early or locally advanced BC patients, who represented a homogenous sample since they maintained the typically expected proportions in terms of BC staging, molecular, and histological subtypes (see demographic results).

With respect to our pilot study, we confirmed the high prevalence of headaches and, in particular, of M among BC patients and the lower prevalence of TTH than in the general population. The high prevalence of M in our population is even more striking, considering that we applied the epidemiological approach suggested by ICHD-3 to preferentially diagnose TTH over probable M.

Overall, our results point out a strict co-existence of M and BC rather than a net beneficial effect. In fact, if we assume a protective role of M on BC development, the prevalence of BC cases in migraineurs should be lower than in NONH, whereas we found a higher rate compared to the normal population. In our previous study, we recruited both early and advanced BC patients and found a higher incidence of M in early rather than in advanced BC [31]. We speculated that the lower incidence of M in advanced BC was an indirect sign of a protective effect, although the dimension of the advanced BC population did not allow for drawing firm conclusions. In the present study, the high prevalence was associated with an earlier age at the diagnosis of BC in both MWOA and MWA patients, mostly in MWA, in which BC occurred about 7 years earlier, compared with NONH or TTH patients. Taken together, these results seem not to confirm the protective effect of M in BC development.

The susceptibility of M for BC seems to be also reinforced by BC characteristics and course. Indeed, we found a stronger link between MWA and BC staging, with a progressive reduction of the chance of being diagnosed with higher stages for NONH and, by contrast, a higher risk of being diagnosed at II or III stages for MWA patients, while the risk to be diagnosed in the III stage was equally higher for MWA and MWOA patients.

BC diagnosis and the therapeutic management of BC (including chemotherapy and hormonal therapy) seemed not to influence headache patterns in both M and TTH groups, except for radiotherapy. MWA patients experienced more changes in their habitual pattern after locoregional radiotherapy. This is an unexpected result that might be related to the higher susceptibility of MWA rather than MWOA to external triggers for attacks since a higher number of MWA experience a worsening of the previous frequency [33]. Interestingly, we also found that M patients exhibited a higher prevalence of side effects due to systemic chemotherapy with taxanes (i.e., peripheral neuropathy) compared to NONH. This finding is quite novel, the taxane-induced neuropathy being devoid of known predictive factors. In this scenario, M could have a potential negative impact on the management of BC treatment. A possible pathophysiological explanation could be that migraine and neuropathies can occur together quite often [34] and also possibly share some pathophysiological mechanisms [35].

To corroborate the complex nature of the interaction between M and BC, we found a direct positive correlation between headache frequency (expressed in headache days per month) and both ER and PgR receptors expression in headache patients. However, when divided according to the headache diagnosis, the correlation persisted only for PgR in MWOA. Although the relationship between M and female hormones has been extensively investigated, the majority of studies pointed towards a main role for estrogens rather than progestins. Recently, it was suggested that the association among estrogens, M and BC, could depend on a common genetic environment predisposing to both M and HR-positive BC. Indeed, an intronic polymorphism (rs2234693) in *ER alpha* was related specifically to MWA [36] and BC when the 325 C > G polymorphism was present [37]. In the present study, the strongest correlation was found with progesterone which is known to favor the release of neuropeptides involved in M mechanisms [38], thus increasing the chances of having an M attack.

In our previous pilot study, we also found that higher ER expression was found in BC patients with an active rather than a prior history of headaches [31]. In this study, by collecting the number of headache days, we succeeded in highlighting a deeper relationship with headache characteristics. This confirmed association between headache frequency and HR positivity strengthens the hypothesis that it could be related to mechanisms involved in the generation of the M attack rather than M per se. Indeed, during an M attack, several pro-inflammatory neuropeptides increase their concentrations [38,39,40]. Both serotonin 5-Hydroxytryptamine receptor 2B (5-HTR2B) and 5-Hydroxytryptamine receptor 4 (5-HTR4) correlate with ER-α and with ER-α and progesterone, respectively [41]. Furthermore, calcitonin gene-related peptide (CGRP) and CGRP receptor are located in the same neurons containing ER [42]. In a recent rodent model of M, authors found that, during the estrous, the level of CGRP was lower, thus suggesting that CGRP might have been released during the drop in estrogens and strengthening the relationship between the two mechanisms [43].

In this study, we did not confirm the previous association between M and HER2-positive BC [31]. The rate of HER2-positive BC was similar in all patients included in the analysis (i.e., MWA, MWOA, TTH, and NONH). To draw firm conclusions on this association, further investigations are needed. Recently, the HER2 positivity classification has been challenged by the notion of the existence of a HER2 low category. Traditionally only patients presenting at least an immunohistochemistry positivity score of 2+ up could be considered HER2 positive and treated with anti-HER2 agents. However, recently some authors raised the hypothesis that patients with lower expression of HER2 (namely HER2 low) could also benefit from specific anti-HER2 therapy [44].

Taken together, the results obtained point towards an interplay of BC and M without a net beneficial effect of M on BC but rather a reciprocal influence of the two conditions mediated by the interplay between M and BC molecular profiles. The mechanisms responsible for this interplay remain largely unknown, possibly involving a complex overlap of hormonal, neurotransmitter, and neuropeptide combinations. Furthermore, the immune system may also play a role since cytokine alterations have been reported in both M and BC, including an imbalance in the T helper 17 (Th17) and regulatory T (Treg) cells [45,46,47,48]. However, before completely ruling out a possible protective effect of M, we have to keep in mind that our findings were raised from a cross-sectional report of a longitudinal study and, at present, the observational time is still too short to demonstrate the actual impact of M on the BC course. To date, less than 10% of our BC patients relapsed.

Although the design of the present study was improved compared to the pilot one, there are still some limitations. One could be that we used a questionnaire-based diagnosis of the headache rather than a method of anamnestic medical history taking, which represents the gold standard. However, this approach is widely used in headache research [49]. Moreover, due to the busy schedule of oncology visits, it was difficult to refer BC patients to an extra neurologic visit, even in the context of a research study. Our choice was supported by the observation in our pilot study that the majority of headache patients who returned the questionnaire were not interested in dealing with their headache, even if they had a bothersome number of headache days or an active headache at the time of recruitment.

Another limitation is that during this study, we observed a limited number of relapsed patients; thus, we cannot properly analyze the actual outcome of BC patients with M compared with that of non-headache patients. We aim to update the results in the following years to pinpoint the role of M in BC relapse.

## 5. Conclusions

In conclusion, M was found to be significantly more prevalent in BC patients compared to the non-headache population. Our findings demonstrated that migraineurs were diagnosed with BC at an earlier age and at more advanced stages compared to those who did not suffer from headaches. Moreover, taxane-induced neuropathy was more prevalent among BC patients with M. Taking into account these findings, M could be considered more as a risk factor rather than a protective factor, as seen in some previous studies. The incidence of M attacks was found to be increased in HR+ BC patients and in particular in MWOA and PgR-positive BC, suggesting that hormonal status could represent the major common field of the two conditions.

## Figures and Tables

**Table 1:** Demographic, clinical, and pathological characteristics of BC patients.

	Patients (*n* = 440)	
	Mean	SD
Age	53.8	12.1
BMI	24.7	4.9
ECOG-PS, median (range)	0	(0–3)
	n	%
Menopausal status		
Premenopause	161	36.6
Postmenopause	242	55.0
Perimenopause	32	7.3
NA	5	1.1
Comorbidities		
Cardiovascular	49	11.1
Metabolic	32	7.3
Cardiovascular + metabolic	14	3.2
Cardiovascular + other	34	7.7
Metabolic + other	30	6.8
Cardiovascular + metabolic + other	36	8.2
Other comorbidities	84	19.1
No comorbidity	161	36.6
Histologic subtype		
NST	347	78.9
Lobular	52	11.8
Other	17	3.9
Mixed	20	4.5
NA	4	0.9
Nottingham score		
G1	26	5.9
G2	271	61.6
G3	132	30.0
NA	11	2.5
Clinical stage		
I	204	46.4
II	191	43.4
III	45	10.2
Molecular Subtype		
Luminal A	187	42.5
Luminal B HER2 negative	127	28.9
Luminal B HER2 positive	60	13.6
HER2 positive	29	6.6
TNBC	36	8.2
HR (HER2 NA)	1	0.2

Abbreviations: *n*, number; SD, standard deviation; BMI, body mass index; ECOG-PS, Eastern Cooperative Oncology Group Performance Score; NST: no special type; NA, not available; HR, hormone receptors; HER2, Human Epidermal Growth Factor Receptor 2; TNBC, triple-negative breast cancer.

**Table 2:** BC patients’ treatments.

	*n*	%
Chemotherapy (n = 207)		
AC	40	19.3
Taxanes	14	6.8
AC + Taxanes	143	69.1
Other	10	4.8
Hormonal therapy (n = 365)		
Tamoxifen	131	35.9
AI	173	47.4
Switch	57	15.6
Other	4	1.1
LH-RH analog		
yes	122	27.7
no	318	72.3
Radiotherapy		
yes	324	73.6
no	116	26.4

Abbreviations: *n*, number; AC, anthracyclines plus cyclophosphamide; AI, Aromatase Inhibitor; LH-RH, Luteinizing Hormone-Releasing Hormone.

**Table 3:** Headache patients’ characteristics.

	Headache Patients (*n* = 319)		
	MWA	MWOA	TTH
Number of patients (%)	59 (18.5)	188 (58.9)	72 (22.6)
Frequency days/month (SD)	4.2 (±7.7)	2.7 (±5.2)	1.7 (±4.1)
Age at onset (SD)	24.8 (±14.1)	23.7 (±12.5)	25.1 (±14.0)
% Menstrually related (% on total headache patients)	50.8 (9.4)	59.6 (35.0)	25.0 (5.8)
% Ongoing preventive therapy (% on total headache patients)	6.8 (1.3)	2.1 (1.3)	2.8 (0.6)

Abbreviations: *n*, number; MWA, migraine with aura; MWOA, migraine without aura; TTH, tension-type headache; SD, standard deviation.

**Table 4:** Headache variation in relation to menopause, BC diagnosis, and therapy.

	MWA%	MWOA%	TTH%
Menopause			
Improved	43.9	41.1	26.1
Worsened	17.5	8.1	5.8
Started	0.0	1.1	0.0
No change	33.3	39.5	66.7
Premenopause	5.3	10.3	1.4
BC diagnosis			
Improved	10.5	3.8	1.4
Worsened	8.8	4.8	15.5
Started	0.0	0.5	0.0
No change	80.7	90.4	83.1
NA	0.0	0.5	0.0
Chemotherapy			
Improved	5.3	3.8	2.9
Worsened	14.0	5.9	10.1
Started	0.0	0.0	0.0
No change	26.3	39.5	34.8
Therapy not carried out	54.4	50.8	52.2
Hormonal therapy			
Improved	8.8	10.9	1.4
Worsened	19.3	12.0	11.4
Started	0.0	0.0	0.0
No change	57.9	62.0	60.0
NA	0.0	0.5	0.0
Therapy not carried out	14.0	14.7	27.1
Radiotherapy			
Improved	3.5	0.0	0.0
Worsened	7.0	2.2	4.4
Started	0.0	0.0	0.0
No change	52.6	74.5	66.2
Therapy not carried out	36.8	23.4	29.4

Abbreviations: MWA, migraine with aura; MWOA, migraine without aura; TTH, tension-type headache; BC, breast cancer; NA, not available.

**Table 5:** Headache diagnosis in relation to clinical stage.

BC Patients (*n* = 435)				
	NONH	MWA	MWOA	TTH
Stage I	57	17	93	36
Stage II	49	36	75	27
Stage III	10	6	20	9

Abbreviations: *n*, number; MWA, migraine with aura; MWOA, migraine without aura; TTH, tension-type headache; NONH, patients without headache.

## Data Availability

The data presented in this study are available on request from the corresponding author. The data are not publicly available due to patients’ privacy.
